# Practice-based initial teacher education in alternative teaching certification programs: A scoping review protocol

**DOI:** 10.1371/journal.pone.0360047

**Published:** 2026-10-13

**Authors:** Gerardo Urra-Barra, Graciela Acevedo, Erich Leighton-Vallejos, Víctor Ruiz-González, Camila Díaz-Ibáñez

**Affiliations:** Facultad de Educación, Universidad San Sebastián, Chile; Universiti Pertahanan Nasional Malaysia, MALAYSIA

## Abstract

Given the shortage of specialized teachers, Initial Teacher Education requires alternative teacher preparation routes, such as Alternative Teaching Certification Programs, that allow the incorporation of professionals who already possess prior work experience into the educational system. However, this situation raises new challenges regarding the design and implementation of the teacher preparation model within these programs. Consequently, this study aims to map and synthesize the existing evidence on the characteristics of practice-based teacher education (PBTE) programs, understood as an approach in which professional learning is constructed through authentic, situated teaching experiences rather than primarily theoretical instruction, designed for individuals who already hold technical, bachelor’s, or professional degrees. The review will be conducted following the JBI (Joanna Briggs Institute) methodology for scoping reviews, and will be reported in accordance with PRISMA-ScR guidelines. Data extraction and synthesis will be structured around the JBI Participants, Concept, and Context (PCC) analytical framework. Database searches will be conducted in Web of Science (WoS) and Scopus, including studies published between 2009 and 2025 in English, Spanish, and Portuguese. The review will specifically examine program duration, mentoring structures, practicum design, institutional type, teaching strategies, and reported outcomes. This protocol has been registered in the Open Science Framework (OSF) and will be publicly available once approved.

## Introduction

Initial Teacher Education (ITE; in Spanish, *Formación Inicial Docente*, FID) is understood as a systematic and structured process aimed at developing the professional competencies required for teaching across the different levels of the educational system [1,2].

At a global level, the United Nations estimate that approximately 44 million new teachers will be needed by 2030 in order to respond to the growing educational demand [3]. This shortage poses concrete demands on teacher preparation systems, which must not only increase enrollment, but also guarantee training that is both high in quality and relevant to current teaching practice.

In this context, alternative teaching certification programs have emerged as a relevant alternative within the Chilean tertiary education system. For the purposes of this review, Alternative Teaching Certification Programs (ATCPs) are defined as initial teacher education pathways designed for individuals who already hold a prior technical, professional, or academic qualification, offering an abbreviated or alternative route to a teaching credential that recognizes prior learning and work experience [4]. These programs connect prior learning experiences with the completion of a bachelor’s degree in education and a specific disciplinary teaching certification. The National Accreditation Commission (CNA; in Spanish: [5] defines these programs as designed, formalized, and implemented with the purpose of offering study continuity to individuals who already hold a prior professional or technical degree or an academic qualification, recognizing work experience and knowledge acquired within the school system. Notwithstanding, their growth also raises challenges regarding the program’s quality assurance, particularly under current regulations, in which accreditation is required to enroll new students.

In response to these challenges, it becomes necessary to reconsider the teacher preparation models offered to pre-service teachers. Within this scenario, the international approach of Practice-Based Teacher Education (PBTE) has gained relevance. PBTE argues that professional learning should be constructed through authentic teaching experiences that integrate pedagogical theory, disciplinary knowledge, and situated action in the classroom [6,7]. This approach implies a substantial transformation in the design of ITE (FID) programs, particularly in relation to mentoring practices and the articulation between universities and schools. Unlike models focused primarily on theoretical or didactic preparation, PBTE promotes the active participation of pre-service teachers in simulated teaching, analysis of real classroom practices, guided mentorship, and systematic processes of critical reflection [8,9]. Its implementation becomes especially relevant in alternative teaching certification programs, where candidates bring prior educational and/or work experiences that require pedagogical proposals capable of recognizing and articulating those forms of knowledge with concrete opportunities for situated and contextualized professional development. While PBTE is associated with stronger practical readiness and greater coherence between theory and classroom action, the literature also identifies persistent tensions regarding the risk of reducing the theoretical and disciplinary foundations of teacher preparation, the resource demands of high-quality mentorship, and the variability in how authentic practice is operationalized across programs and institutional contexts.

Despite its expansion, the ways in which this approach is implemented to guide pedagogical processes in teacher education programs vary significantly across countries, institutions, and levels of development of teacher preparation systems [10–13]. These experiences illustrate that PBTE is being adopted and adapted through diverse methodological approaches, without yet converging on shared definitions, common program structures, or agreed criteria for what constitutes a practice-based component in teacher preparation. This conceptual and empirical heterogeneity signals a field that is still under development, for which a scoping review is the most appropriate first synthesis step rather than a systematic effectiveness review.

Given the diversity of frameworks, designs, and contexts involved, a scoping review was considered the most appropriate methodological approach. Unlike systematic reviews focused on effectiveness, scoping reviews allow researchers to explore the range, nature, and characteristics of a broad body of literature, map emergent categories, and synthesize key concepts [14,15]. This approach is particularly pertinent when the field of study is under development, as is the case of PBTE, and when a structured overview of the available knowledge is required.

For this reason, the purpose of this scoping review is to map the available literature on PBTE, identifying its main characteristics in terms of program duration, mentoring structures, practicum design, and institutional type, as well as pedagogical strategies and reported outcomes, in the context of alternative teaching certification programs that enable professionals with prior educational experiences and certifications to obtain a teaching degree.

### Research question

What evidence does the literature report regarding the design, implementation, and outcomes of practice-based initial teacher education programs for individuals with existing degrees, including post-baccalaureate, alternative, and consecutive routes?

## Methods

The proposed scoping review will be conducted in accordance with the Joanna Briggs Institute (JBI) methodology for scoping reviews, as outlined in the JBI Manual for Evidence Synthesis [14], and following the PRISMA-ScR recommendations [16]. The protocol has been registered in the Open Science Framework (OSF) under the registration number 10.17605/OSF.IO/UTR6V, ensuring transparency and traceability throughout the process.

### Inclusion criteria

Participants: Studies involving both teacher candidates and teacher educators participating in programs explicitly defined by their authors as alternative teaching certification programs, and that are directed at students or professionals who hold a prior academic or professional qualification (e.g., bachelor’s degree, tertiary technical degree, or professional title) will be included. Studies reporting only on traditional initial teacher education programs (i.e., those involving teacher candidates without such prior qualifications) will be excluded.

Concept: Studies examining initial teacher education programs that implement a practice-based teacher education (PBTE) approach will be considered. PBTE is characterized by the structured and systematic integration of authentic teaching experiences as the core element of teacher professional learning. Such experiences include, among others, simulated lessons, analysis of real classroom practice, reflective practice, and guided mentorship provided by experienced educators [6,17]. For the purposes of this review, a program will be considered PBTE-aligned if it explicitly incorporates at least one of the three processes described by Grossman et al. [8]: representation of practice, decomposition of practice, or approximation to practice, implemented in a structured and recurring manner within the program design. No restrictions will be placed regarding the length of the program, disciplinary area, intended education level, or any other characteristics of the program or its participants. The language of program implementation is not a restriction; however, publications reporting on the program must be written in English, Spanish, or Portuguese, as these are the languages the research team can assess.

Context: Studies conducted in practice-based initial teacher education programs directed at individuals with prior academic or professional qualifications (post-baccalaureate/alternative/consecutive routes) and delivered by higher education institutions worldwide will be included.

### Types of sources

This scoping review will consider studies adopting experimental and quasi-experimental designs. Likewise, analytical observational studies including prospective and retrospective cohort studies, case-control studies, and analytical cross-sectional studies will be included. Descriptive observational designs such as case series, single case reports, and descriptive cross-sectional studies will be considered.

Additionally, qualitative studies informed by approaches such as phenomenology, grounded theory, ethnography, qualitative description, and action research will be included. Opinion pieces and reflective articles relevant to the object of study and meeting the inclusion criteria will also be considered. Opinion and reflective articles are included because PBTE within alternative teaching certification programs is an emerging area in which much of the available knowledge is still expressed through expert commentary and practitioner reflection rather than empirical studies. During data charting, these sources will be flagged by type and synthesized separately from empirical studies in the narrative synthesis.

### Search strategy

Systematic database searches will be conducted in Web of Science Core Collection and Scopus. These two databases were selected because they offer the broadest interdisciplinary coverage of peer-reviewed literature in education and social sciences, support advanced Boolean search logic, and together index the large majority of high-impact journals in teacher education research [18,19]. The search strings were structured in three thematic blocks: Block A covers PBTE terminology, Block B covers initial teacher education, and Block C covers alternative certification programs. The three blocks are crossed using the AND operator. No language restriction is applied at the level of program implementation; studies are limited to those published in English, Spanish, or Portuguese, reflecting the linguistic competencies of the research team. Studies published between 2009 and 2025 will be included, as this period corresponds to the emergence and consolidation of practice-based approaches in teacher education, particularly with the development of models such as practice-based teacher education and the consolidation of concepts such as core practices and high-leverage practices [7,8]. The complete Boolean search strategies for both databases are presented in Table 1 (Web of Science) and Table 2 (Scopus).

**Table 1:** Web of Science Core Collection search strategy (TS = Topic field).

#	Search query
1	TS=(“practice-based teacher education” OR “practice-based teacher preparation”)
2	TS=(“core practices” OR “high-leverage practices” OR “high leverage practices”)
3	TS=(“pedagogies of enactment” OR “approximations of practice” OR “decompositions of practice”)
4	TS=(practicum OR “field experience” OR “clinical experience” OR “mentored teaching” OR “guided mentorship”)
5	#1 OR #2 OR #3 OR #4 [Block A — PBTE]
6	TS=(“initial teacher education” OR “teacher preparation” OR “teacher training” OR “preservice teacher” OR “pre-service teacher”)
7	TS=(“student teacher” OR “teacher candidate” OR “prospective teacher” OR “teacher educator”)
8	#6 OR #7 [Block B — ITE]
9	TS=(“alternative certification” OR “alternative licensure” OR “alternative route” OR “alternative teacher certification”)
10	TS=(“post-baccalaureate” OR postbaccalaureate OR “consecutive teacher education” OR “consecutive route”)
11	TS=(“career changer” OR “second-career teacher” OR “transition-to-teaching”)
12	#9 OR #10 OR #11 [Block C — ATCP]
13	#5 AND #8 AND #12 [Final intersection]
14	#13 AND Publication Years: 2009–2025

Source: Prepared by the authors.

**Table 2:** Scopus search strategy (TITLE-ABS-KEY field).

#	Search query
1	TITLE-ABS-KEY(“practice-based teacher education” OR “practice-based teacher preparation”)
2	TITLE-ABS-KEY(“core practices” OR “high-leverage practices” OR “high leverage practices”)
3	TITLE-ABS-KEY(“pedagogies of enactment” OR “approximations of practice” OR “decompositions of practice”)
4	TITLE-ABS-KEY(practicum OR “field experience” OR “clinical experience” OR “mentored teaching” OR “guided mentorship”)
5	#1 OR #2 OR #3 OR #4 [Block A — PBTE]
6	TITLE-ABS-KEY(“initial teacher education” OR “teacher preparation” OR “teacher training” OR “preservice teacher” OR “pre-service teacher”)
7	TITLE-ABS-KEY(“student teacher” OR “teacher candidate” OR “prospective teacher” OR “teacher educator”)
8	#6 OR #7 [Block B — ITE]
9	TITLE-ABS-KEY(“alternative certification” OR “alternative licensure” OR “alternative route” OR “alternative teacher certification”)
10	TITLE-ABS-KEY(“post-baccalaureate” OR postbaccalaureate OR “consecutive teacher education” OR “consecutive route”)
11	TITLE-ABS-KEY(“career changer” OR “second-career teacher” OR “transition-to-teaching”)
12	#9 OR #10 OR #11 [Block C — ATCP]
13	#5 AND #8 AND #12 [Final intersection]
14	#13 AND PUBYEAR > 2008 AND PUBYEAR < 2026

Source: Prepared by the authors.

### Selection of studies and sources of evidence

All citations will be imported into Rayyan, a systematic screening tool, and duplicates will be removed automatically. Prior to full-scale screening, a pilot test will be conducted with four articles selected purposively from the initial search results to represent a range of study designs and geographic contexts. The pilot aims to confirm inter-rater agreement on the application of the eligibility criteria and to identify any ambiguity in their wording. An agreement rate below 75% on the pilot set will trigger a joint discussion between reviewers and, if necessary, a revision of the inclusion and exclusion criteria before full screening proceeds. It is acknowledged that four articles constitute a limited pilot sample given the conceptual complexity of PBTE; the research team will therefore remain attentive to recurring disagreements during the early stages of full screening as an informal extension of the pilot phase. After the pilot, titles and abstracts will be assessed independently by at least two reviewers. References that meet the inclusion criteria will proceed to full-text assessment, which will also be conducted independently by two reviewers. Any discrepancies will be resolved by consensus or by involving a third reviewer.

The complete study selection process will be reported in the final review using a PRISMA-ScR flow diagram for scoping reviews [20].

### Data extraction

Data will be extracted independently by two or more reviewers using a data extraction tool specifically designed by the research team for this review. This tool will include structured fields aligned with the PCC framework (Participants, Concept, and Context). Furthermore, this tool will also include additional variables concerning methodological designs, program implementation characteristics, and the main findings reported in each study. This strategy will ensure a systematic, rigorous, and coherent data collection process aligned with the objectives of the scoping review.

The data extraction form will be piloted with an initial sample of included studies to assess its relevance, clarity, and alignment with the review objectives. Based on the pilot, the research team will refine the form as necessary before proceeding with full data extraction. Any modifications made to the extraction tool during the review process will be documented and justified in the final review to ensure transparency and reproducibility.

In cases where relevant information is not available in the full text of a study, corresponding authors will be contacted to request missing data or necessary clarifications. If no response is received within two weeks, a reminder will be sent. If the information remains unavailable after a further two weeks, the missing data will be coded as not reported. If the search yields fewer studies than anticipated, the research team will broaden the search terms and extend manual reference list screening before concluding that the evidence base is genuinely limited. If a database becomes temporarily inaccessible during the search window, the search for that database will be repeated when access is restored and the execution date will be documented in the PRISMA-ScR flow diagram.

### Data analysis and presentation

The results will be presented descriptively and narratively, organized around the components of the PCC framework and directly addressing the objectives and central research question of the review. Accordingly, different formats will be used to enhance the visualization and understanding of the findings.

First, the results will be organized into tables that will map key characteristics of the included studies, such as country of origin, type of program, participant population, type of institution, methodological approach, implemented practice-based components, and main findings. These tables will provide an overview of how practice-based teacher education has been addressed in programs aimed at individuals with prior technical, academic, or professional qualifications.

Second, graphical representations and thematic maps will be used to identify patterns, knowledge gaps, and geographical or institutional concentrations within the available evidence. These visualizations will support the identification of emerging areas, recurrent practices, and potential gaps in the implementation of the PBTE approach across specific contexts.

In addition, a narrative synthesis will be conducted to interpret the findings in relation to the PCC framework. Themes will be identified using a primarily deductive approach structured around the dimensions stated in the research objective, specifically program duration, mentoring structures, practicum design, and institutional type, while remaining open to inductively coding patterns that emerge from the data and do not fit these predefined categories. Two reviewers will independently group the extracted data into preliminary themes. Discrepancies in theme labeling or grouping will be resolved by discussion and, where necessary, by a third reviewer.

Finally, in accordance with the purposes of a scoping review, no critical appraisal of methodological quality will be conducted for the included studies. However, when appropriate, comments will be made regarding the diversity of designs and methodologies used, with the aim of informing future systematic or empirical research.

### Significance

At the national level, this review responds directly to the expansion of alternative teaching certification programs in Chile under accreditation requirements that currently lack an evidence base on how practice-based components should be designed and implemented. Mapping international evidence on PBTE can therefore inform program design and quality assurance criteria in this specific context. At the global level, given the United Nations projection of approximately 44 million additional teachers needed by 2030 [3], alternative certification routes are expanding as a workforce strategy across multiple countries. This review contributes by synthesizing, for the first time, evidence on how practice-based approaches have been conceptualized and implemented specifically within this type of program, supporting future systematic reviews and informing policy decisions in national systems facing comparable teacher shortage pressures.

### Limitations

This protocol presents several anticipated limitations. First, restricting the systematic database search to Web of Science and Scopus, while justified by their interdisciplinary coverage and advanced Boolean search capabilities, means that studies indexed exclusively in discipline-specific or regional databases may not be captured. Second, the pilot test involves only four articles, which is a modest sample given the conceptual complexity of PBTE; this is acknowledged and partially mitigated by the informal extension of the pilot phase during early full-text screening. Third, the inclusion of opinion and reflective articles alongside empirical studies introduces heterogeneity in the strength of evidence synthesized; this is addressed by analyzing and reporting these source types separately in the narrative synthesis. Fourth, consistent with standard scoping review methodology, no formal quality appraisal of included studies will be conducted; the review will therefore map the breadth of available evidence rather than evaluate its methodological robustness. Fifth, while the regulatory context of Chilean alternative teaching certification programs motivates this study, the global scope of the review means that findings will need to be interpreted in light of each country’s specific institutional and regulatory conditions.
